# Physiological Signatures of Emotional Processing via Pupil Diameter, Galvanic Skin Response, and Gaze Behavior: A Pilot Study

**DOI:** 10.3390/jemr19040072

**Published:** 2026-07-07

**Authors:** Miwa Horiuchi-Hirose

**Affiliations:** Department of Health and Nutrition, Tokiwa University, Mito 310-8585, Ibaraki, Japan; m-hirose@tokiwa.ac.jp

**Keywords:** eye tracking, trait anxiety

## Abstract

Background: This pilot study investigated the effects of emotional images on gaze behavior, pupil diameter, and galvanic skin response (GSR). After measuring trait anxiety, 37 healthy adults were shown images (disgust, fear, happiness, sadness, or neutral) from the International Affective Picture System for 4 s each while recording eye tracking and physiological responses. Methods: Recorded data included total fixation duration, average fixation duration, fixation count, first fixation duration, mean pupil diameter during fixation, and average GSR. Results: Compared to images depicting happiness, those depicting fear were associated with longer average fixation duration, fewer fixations, and larger pupil diameters. Compared with neutral images, those showing happiness were associated with larger pupil diameters. No significant differences in GSR were observed across emotion categories. Regarding anxiety levels, fear-inducing images tended to be associated with fewer fixations and larger pupil diameters across all anxiety groups. In moderate- and high-anxiety groups, there was a tendency for longer fixation duration compared to images evoking happiness. Conclusions: Overall, these preliminary findings suggest that while individuals tend to fixate on fear images less often than on happiness images, they fixate for longer on them, which may cause pupil dilation. However, no significant response was observed depending on the level of trait anxiety. Further research using a larger sample size is needed to confirm these initial observations.

## 1. Introduction

Objectively assessing an individual’s anxiety allows implementing appropriate interventions and the exploration of approaches to improve mental health. Despite trait anxiety, a relatively stable indicator of an individual’s tendency to experience anxiety, being a widely used indicator in research [1], objectively capturing trait anxiety remains challenging. For instance, eye-tracking research has shown that individuals with high anxiety levels exhibit a pronounced attentional bias toward threatening stimuli [2]. Furthermore, during free-viewing tasks, depressed individuals show decreased attention and shorter fixation durations on positive stimuli, with increased fixation durations on aversive stimuli, rather than an increased vigilance toward threats [3]. Furthermore, emotional responses are accompanied by alterations in autonomic nervous system activity; pupil diameter and galvanic skin response (GSR) are commonly used as physiological indicators [4]. Pupil diameter dilates in preparation for escape or avoidance behavior [5], reflecting surprise [6], and serving as an arousal index [3]. Pupillary dilation is instructed through various sympathetic pathways, involving efferent projections from the locus coeruleus to regions such as the hypothalamus, amygdala, and cerebral cortex [7,8]. These pupillary reactions are elicited not only by fear-inducing images [9], but also by both pleasant and unpleasant images [10]. Meanwhile, GSR reflects increased skin conductance resulting from elevated sweat secretion from eccrine glands; this process is modulated by the sympathetic nervous system, which is controlled by the hypothalamus and associated with brain regions such as the anterior cingulate cortex, insula, and ventral prefrontal cortex [11,12]. GSR also increases in response to both pleasant and unpleasant images [10]. In the present study, we focused on gaze behavior, pupil diameter, and GSR to investigate responses to emotional stimuli.

To induce a state of anxiety, we took advantage of the International Affective Picture System (IAPS), which can elicit emotional arousal. This choice was based on findings that IAPS images inducing negative emotions (disgust, fear, and sadness) are primarily associated with the occipitotemporal cortex and the amygdala–hippocampal complex [13]. Furthermore, fear stimuli have been reported to evoke extensive activation from the visual association cortex to the temporal lobe and fusiform gyrus [14]. In contrast, sadness stimuli are associated with the activation of the medial superior frontal cortex, cerebellum, and visual cortex [14]. Collectively, these findings demonstrate that distinct neural substrates underlie different emotions. Accordingly, we formulated the following hypotheses. First, fear-inducing stimuli will cause pupil dilation, increased GSR, and changes in fixation duration and frequency. Second, when processing such threat-related stimuli, participants with high trait anxiety will exhibit heightened sympathetic nervous system activity while modulating their gaze behavior and changing the temporal dynamics of attentional allocation.

In this pilot study, we used trait anxiety to conduct exploratory research into the synchronous dynamics of the physiological responses that occur during processing of specific emotional categories (namely, disgust, fear, happiness, and sadness).

## 2. Materials and Methods

### 2.1. Participants

Participants were university students recruited via flyers posted on the Tokiwa University campus. The final sample was 37 participants (mean age = 19.57 years, standard deviation = 0.90), comprising 27 females and 10 males. Participation was limited to individuals whose native language was Japanese, who had uncorrected or corrected visual acuity of ≥1.0, who could view a laptop screen without difficulty, and who did not have strabismus. Prior to participation, the purpose and procedures of the study were explained and informed consent was obtained. This study was approved by the Tokiwa University Ethics Committee (Approval No.: 100172). Participants were provided with a predetermined honorarium.

### 2.2. Stimuli

Photographs from the IAPS were used as stimuli. This study utilized images that have been shown to elicit brain responses to landscape stimuli not only in healthy individuals, but also in patients with schizophrenia. The images were classified into five emotional categories: disgust, fear, happiness, sadness, and neutral. A total of 25 standardized images were selected from the International Affective Picture System (IAPS), comprising 5 images for each of the five emotional categories (disgust, fear, happiness, sadness, and neutral). To control for potential carryover and order effects, the presentation order of the images was completely randomized for each participant. Specifically, a random number generator via the RAND function in Microsoft Excel was utilized to construct a unique, randomized sequence of stimuli for every subject. All participants viewed the exact same set of 25 images to ensure consistency across the sample. The mean valence and arousal values were as follows: disgust (valence = 2.7, arousal = 5.3), fear (valence = 3.3, arousal = 6.2), neutral (valence = 5.2, arousal = 3.0), sadness (valence = 2.7, arousal = 4.7), and happiness (valence = 7.5, arousal = 4.6). The images were the same as those used by Garcia-Leon et al. [14]. Although the overall analysis revealed a significant difference in luminance across categories, multiple pairwise comparisons showed a significant difference only between fear images and disgust images.

### 2.3. Procedure

At the start of the experiment, participants completed the State-Trait Anxiety Inventory (STAI) as a measure of trait anxiety [15]. For skin conductance measurement, probes were attached to the middle and ring fingers of the participant’s dominant hand. After a 10 min rest period, instructions for the experiment were presented on a monitor and participants were asked to read them carefully. Eye-tracking calibration was then performed, followed by the sequential presentation of stimulus images. The experiment was performed with participants seated in a chair with both elbows resting on a desk. Images were viewed from approximately 60 cm from the monitor while minimizing facial movements.

In each trial, five types of emotional stimulus images (disgust, fear, happiness, sadness, and neutral) were displayed for 4 s each (1 block = 5 images total). Following the presentation of one block, three symbolic stimuli were presented for 4 s each to serve as a washout. The three symbolic stimuli used during this washout period were black crosses (+), (&), and (%), presented in the center of a gray background. These were designed to minimize visual complexity, and their average luminance and contrast were matched (normalized) to the average values of all IAPS images in the main study.

### 2.4. Eye Tracking and Skin Conductance Apparatus

Eye tracking was performed using a Tobii Pro Fusion eye tracker (Tobii AB, Stockholm, Sweden, sampling rate = 60 Hz) connected to a 27” laptop. This device has been widely used in previous studies [16]. Tobii Pro Lab software (Version 1.207) was used for data recording and analysis, and a 9-point calibration was performed. The experiment was conducted in a room shielded from direct sunlight, with fluorescent lighting used to ensure consistent lighting conditions. Skin conductance was assessed using a Shimmer3 GSR+ unit. This device measures the conductivity between electrodes attached to two fingers and has been shown to be a valid indicator of stress [17,18]. The acquired data was imported into the Tobii Pro Lab software. The GSR data were analyzed using preprocessed values. To minimize the confounding effects of significant inter-subject variability in baseline skin conductance and reactivity, the data were converted to within-subject Z-scores.

### 2.5. Statistical Analysis

Statistical analysis was performed using IBM SPSS Statistics Ver. 29. The following metrics were extracted from the gaze data: average fixation duration, mean pupil diameters during fixation, first fixation duration, fixation count, and total duration of whole fixations. The GSR metric used was the average GSR. First, a repeated-measures analysis of variance (ANOVA) was performed for each variable, with emotional category (disgust, fear, happiness, sadness, and neutral) as the factor. The Bonferroni method was applied for subsequent multiple comparisons, with *p* < 0.05 considered statistically significant.

Based on their STAI scores, participants were classified into a low trait anxiety group (STAI score < 40 for men, <43 for women), moderate trait anxiety group (STAI score 40–52 for men, 43–51 for women), and a high trait anxiety group (STAI score ≥ 53 for men, ≥50 for women) [15]. We then performed a two-way repeated-measures ANOVA with trait anxiety level (low, moderate, and high) and emotional stimulus category (disgust, fear, happiness, sadness, and neutral) as factors. The Bonferroni method was applied for subsequent multiple comparisons.

## 3. Results

### 3.1. Effects of Emotional Categories

**Figure 1:**
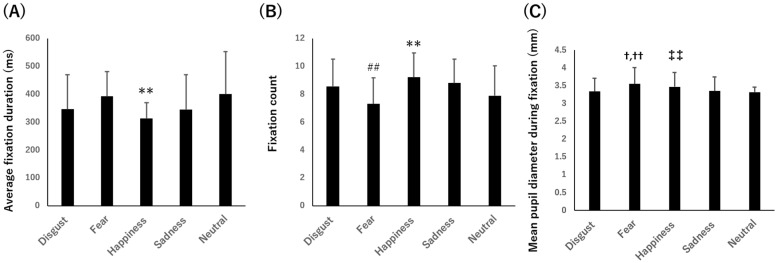
Comparison of eye-tracking metrics and peripheral autonomic responses across five emotional categories. (**A**) Average fixation duration, (**B**) fixation count, and (**C**) mean pupil diameter during fixation. Data is presented as mean ± standard deviation of the mean (SD). Statistical significance is denoted as follows: **: *p* < 0.01, Happiness vs. Fear or Neutral; ##: *p* < 0.01, Fear vs. Disgust, Happiness, or Sadness; †: *p* < 0.05, Fear vs. Happiness; ††: *p* < 0.01, Fear vs. Happiness, Neutral, Sadness, or Disgust; ‡‡: *p* < 0.01, Happiness vs. Neutral, Sadness, or Disgust.

**Figure 2:**
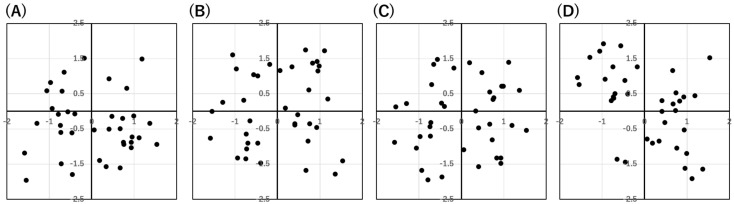
Scatter plot of individual standardized GSR scores across emotion categories against the neutral baseline. The Z-scored galvanic skin response (GSR) of individual participants was plotted with (**A**) disgust; (**B**) fear; (**C**) happiness; and (**D**) sadness on the vertical axis and the neutral condition on the horizontal axis.

Table 1 and Figure 1 present the eye-tracking and Table 2 and Figure 2 present GSR results for the five emotional categories. The ANOVA results showed no significant differences in the total fixation duration, first fixation duration, or average GSR across the emotional categories (disgust, fear, happiness, sadness, and neutral).

Although no significant differences were found in the total fixation duration or first fixation duration, the eye-tracking metrics tended to exhibit emotional modulation. After adjusting for degrees of freedom using the Greenhouse–Geisser test (F (2.7, 98.8) = 5.81, *p* < 0.001, $\eta_{p}^{2}$ = 0.140), the results showed that happiness images had a significantly lower average fixation duration than fear and neutral images (*p* < 0.001). Analysis of the fixation count based on Mauchly’s sphericity test (W = 0.660, *p* = 0.000, $\eta_{p}^{2}$ = 0.228) and Bonferroni’s multiple comparison test revealed that happiness images resulted in significantly more fixations than fear and neutral images (*p* < 0.001). Fear images had significantly fewer fixations than sadness and disgust images (*p* < 0.001). The results of the analysis based on Mauchly’s sphericity test for mean pupil diameters during fixation were (W = 0.726, *p* = 0.000, $\eta_{p}^{2}$ = 0.426). As shown by Bonferroni’s multiple comparison test, fear images had significantly higher mean pupil diameters during fixation than happiness images (*p* < 0.05) and other categories (*p* < 0.001). In addition, happiness images had significantly higher mean pupil diameters during fixation than disgust, sadness, and neutral images (*p* < 0.001).

To control for substantial inter-individual variability in baseline electrodermal activity, the raw GSR data were normalized using a within-subject Z-score transformation. A repeated-measures ANOVA conducted on these normalized scores, however, revealed no statistically significant main effect of emotional condition on GSR. To further examine the absence of significant main or interaction effects in the GSR, we conducted an individual response analysis in which the Z-scored GSR values for each emotion category were plotted against a neutral baseline (Figure 2). As can be seen visually in the scatter plot, data points were widely and spherically distributed across all four quadrants for all conditions: disgust (A), fear (B), happiness (C), and sadness (D).

### 3.2. Differences in Trait Anxiety Scores

Table 3 and Table 4 present the eye-tracking metrics and GSR data for the five categories of emotional stimuli separately for the low-anxiety, moderate-anxiety, and high-anxiety groups. The mean STAI score for all participants was 47.4 ± 10.1. The mean STAI score was 34.1 ± 3.5 for the low-anxiety group (n = 8), 43.9 ± 2.7 for the moderate-anxiety group (n = 14), and 57.9 ± 4.4 for the high-anxiety group (n = 15).

The two-way repeated-measures ANOVA showed no significant interaction between trait anxiety level and emotional category for GSR or any of the eye-tracking measures. Furthermore, no significant main effects were observed for total duration of whole fixations, first fixation duration, or average GSR. However, there was a significant main effect of emotional category for the average fixation duration, fixation count, and mean pupil diameters during fixation. For the average fixation duration, the main effect was significant after adjusting for degrees of freedom using the Greenhouse–Geisser test (F (2.7, 136) = 5.61, *p* < 0.001, $\eta_{p}^{2}$ = 0.142). The results of the Bonferroni multiple comparison test showed that, in both the high-anxiety and moderate-anxiety groups, the average fixation duration for fear stimuli was significantly longer than that for happiness stimuli (*p* < 0.05). Additionally, analysis based on Mauchly’s sphericity test revealed a significant main effect of the fixation count (W = 0.658, *p* = 0.000, $\eta_{p}^{2}$ = 0.224). Bonferroni’s multiple comparison test revealed that the fixation count for fear stimuli was significantly lower than that for happiness in the three anxiety groups (*p* < 0.05). Within the moderate-anxiety group, the fixation count for fear stimuli was significantly lower than that for sadness stimuli (*p* < 0.05). A significant main effect was also observed for the mean pupil diameters during fixation (W = 0.741, *p* = 0.000, $\eta_{p}^{2}$ = 0.458). Subsequent tests revealed the following findings for each anxiety subgroup. Within the three-anxiety group, pupil diameter was significantly larger for fear stimuli than for neutral, sadness, or disgust (*p* < 0.05) stimuli. In the low-anxiety group, the pupil diameter for happiness stimuli was also significantly larger than that for neutral stimuli (*p* < 0.05).

## 4. Discussion

This pilot study examined the effects of five emotional categories—disgust, fear, happiness, sadness, and neutral—on eye-tracking metrics, pupil diameter, and GSR. Although no significant differences were observed between emotional categories for the total fixation duration, first fixation duration, and average GSR, significant differences were observed for the average fixation duration, fixation count, and mean pupil diameters during fixation. Furthermore, when individual differences in trait anxiety were taken into account, the results suggested that for individuals with moderate to high levels of trait anxiety, fear-inducing stimuli were likely to elicit sustained attention, but no association with physiological responses was observed.

When focusing on the influence of emotion categories alone, contrasting visual search patterns and physiological responses were observed between happiness and fear stimuli. Fear images resulted in significantly higher average fixation durations and pupil diameters than happiness images; happiness images produced larger pupil diameters than neutral images. Bradley et al. [10] reported that pupil diameter increased more when viewing pleasant and unpleasant IAPS images than when viewing neutral ones, with no significant difference between pleasant and unpleasant images. Here, we found that pupil diameter increased significantly more in response to fear and happiness images than to neutral images. For happiness images, the average fixation duration was significantly shorter and the number of fixations significantly higher compared to fear and neutral images. This suggests a tendency to quickly scan the entire image in response to positive stimuli rather than fixating on a single point for an extended period. However, the results showed that, despite a significantly lower number of fixations on fear images, the average pupil diameter was significantly larger than in all other emotional categories. Based on this, it is possible that participants suppressed extensive exploratory behavior (number of fixations) in response to fear stimuli while simultaneously directing intense attention toward specific threats, which rapidly increased their physiological arousal levels.

Next, when examining the effect of trait anxiety levels, no statistical interaction was found between trait anxiety levels and emotion categories; the overall response trends were similar regardless of anxiety level. However, a detailed comparison within each anxiety group revealed interesting differences that partially support previous findings. Specifically, only in the moderate- and high-anxiety groups was the average gaze duration for fear stimuli significantly longer than that for happiness stimuli. This might indicate a tendency toward attention bias. Individuals with higher anxiety levels are likely to fixate on fear stimuli once their gaze is directed there and find it difficult to shift their focus. The fact that this significant difference was not observed in the low-anxiety group suggests that differences in anxiety levels may influence differences in the ability to exercise cognitive control over threats.

Despite the significant difference observed in pupil diameter in response to fear and happiness stimuli, no significant difference was observed in GSR. Bradley et al. [10] reported that skin conductance increased significantly when viewing pleasant and unpleasant photos compared to neutral photos. The arousal level of the pleasant stimuli used by Bradley et al. was 5.5 (vs. 4.6 in the present study) and for unpleasant stimuli was 5.9 (vs. 6.2 in the present study). Despite the higher arousal rating of the unpleasant IAPS images used in our study, no difference in skin conductance was observed. Pupillary dilation is directly linked to a rapid visual threat detection mechanism mediated by the locus coeruleus–norepinephrine system and has very high temporal resolution [10]. This likely allowed us to capture attenuation-driven pupillary modulation, even with the short stimulus presentation times used in this study. In contrast, GSR has a very slow physiological time course, requiring a latency of several seconds before onset or peak [19]. Therefore, while the short stimulus presentation time in this study was sufficient to capture pupillary fluctuations reflecting rapid attention and threat processing, it may not have elicited sustained sympathetic activity (GSR). Liina Juuse (2024) et al. [20] reported a minimal galvanic skin response to fear stimuli. However, the high interindividual variability inherent in skin conductance data may further obscure this emotional status. Crucially, the observed discrepancy between pupil diameter and GSR in the present study may stem not only from high interindividual variability, but also from differences in their underlying neural pathways and temporal dynamics. Therefore, despite these findings on the neural correlates of individual autonomic indicators, the precise mechanisms whereby this frontal–subcortical network differentially controls multiple peripheral autonomic responses simultaneously during brief emotional exposure remain largely unknown. Given the preliminary nature of our pilot data, further research is needed to fully understand how these different physiological reactions dissociate or converge under emotional stress.

If feasible, measurements of pupil diameter might serve as a complementary physiological marker to capture immediate, threat-related attentional reactivity rather than as a definitive diagnostic tool for anxiety levels. Please note that our study did not directly obtain neurophysiological or central nervous system measurements. Thus, these frontal–subcortical mechanisms cannot be definitively concluded from our data; rather, they serve as a framework for our working hypotheses. Although our findings of peripheral physiological modulations are consistent with these neural models, further multimodal research incorporating direct neural measurements is required to validate these specific links. Our lack of significant interactions prevents any definitive conclusions regarding trait anxiety groups. Individuals with high trait anxiety are characterized by a marked tendency for sustained attention, particularly toward negative stimuli [21]. Although a positive correlation between trait anxiety and state anxiety has been reported [22], it has also been reported that fear stimuli may not affect physiological responses such as heart rate, brain waves, and pupil diameter [9]. Since this study also found no significant correlation between trait anxiety and fear stimuli, we believe it is difficult to identify physiological responses specific to each level of trait anxiety.

## 5. Limitations

First, the eye-tracking device used had a sampling rate of 60 Hz—too low to accurately measure the duration of the first fixation at high resolution. In future studies, an eye-tracking device with a higher sampling rate should be used instead.

This study evaluated global eye-tracking metrics across the entire image rather than defining specific regions of interest (ROIs). Since emotional images contain diverse visual layouts and compositions, the observed differences in fixation duration and count might partially reflect the structural configuration of the images rather than pure emotional processing. This methodological constraint limits our ability to isolate top-down emotional attention from bottom-up visual saliency. Future studies should implement rigorous ROI-based analyses targeting specific emotional focal points (e.g., faces or threat-related objects) to better control visual composition effects.

Although we observed no significant differences in average luminance between the main comparison groups in this study (fear vs. neutral, and fear vs. sadness), a significant difference in luminance was observed between the fear and disgust categories. Furthermore, this pilot study did not strictly control for other low-level visual features, such as spatial frequency, contrast, or color saturation, across all emotional categories. Therefore, we cannot completely rule out that the pupillary light reflex or bottom-up visual saliency partially contributed to the observed differences in pupillary responses, particularly between specific negative emotions. This lack of comprehensive physical stimulus control represents a methodological constraint. Future studies need to adopt rigorous stimulus normalization methods or algorithms to ensure that luminance, contrast, and all other low-level visual characteristics are perfectly balanced across emotion categories.

Although we applied within-subject normalization (Z-score transformation) to GSR values to reduce individual baseline variability, the expected changes in skin conductance associated with arousal were not significant. In future studies, it will be necessary to consider measures such as prescreening participants’ skin electrical responsiveness to more reliably capture subtle emotional states.

This study analyzed trait anxiety as a categorical variable to facilitate distinct group comparisons aligned with conventional clinical frameworks. However, this dichotomization leads to a loss of statistical variance and may obscure nuanced, nonlinear relationships that could be captured if anxiety was treated as a continuous variable. Group stratification based on trait anxiety resulted in a small sample size for the low-anxiety group (n = 8). Consequently, statistical power might have failed to detect subtle interaction effects in the repeated-measures analysis of variance. This limitation increases the probability of Type II errors, so the current findings remain preliminary. Furthermore, the sample was heavily skewed toward female participants, which limits the generalizability of our findings, as sex differences in emotional reactivity and anxiety-related attentional biases are well documented.

Finally, because this exploratory pilot study involved multiple pairwise post hoc comparisons across various emotional categories and eye-tracking metrics, there is an inherent risk of in-creasing the Type I error rate. Bonferroni corrections were strictly applied within each specific ANOVA post hoc analysis. However, given the cumulative exploratory nature of the study, these findings should be interpreted as preliminary trends. Future confirmatory studies should preregister specific hypotheses and implement rigorous multiple-comparison corrections to control for false positives. This pilot study examined the effects of five emotional categories—disgust, fear, happiness, sadness, and neutral—on eye-tracking metrics, pupil diameter, and GSR. Although no significant differences were observed between emotional categories for the total fixation duration, first fixation duration, and average GSR, significant differences were observed for the average fixation duration, fixation count, and mean pupil diameters during fixation.

## 6. Conclusions

These preliminary findings indicate that pupil diameter and gaze metrics could vary across emotional categories. Within this sample, fear stimuli showed a tendency toward longer average fixation times and larger pupil diameters than happiness stimuli. Additionally, these results indicate that further investigation is required to clarify how the processing of fear stimuli is modulated by trait anxiety.

## Figures and Tables

**Table 1:** The eye-tracking results for the five emotional categories.

	Disgust			Fear			Happiness			Sadness			Neutral			Significant Comparisons
Total fixation duration	2668.30	±	452.26	2537.57	±	463.56	2717.23	±	373.89	2758.14	±	317.37	2629.85	±	406.08	*n.s.*
Average fixation duration	346.56	±	123.78	392.02	±	88.45	313.38	±	55.66 **	345.69	±	124.22	400.48	±	151.59	Happiness < Fear or Neutral
Fixation count	8.56	±	1.96	7.30	±	1.87 ##	9.21	±	1.76 **	8.80	±	1.73	7.90	±	2.15	Happiness > Fear or Neutral Fear < Sadness, or Disgust
First fixation duration	269.04	±	111.80	311.61	±	141.08	233.05	±	56.65	279.51	±	196.49	308.88	±	172.30	*n.s.*
Mean pupil diameter during fixation	3.34	±	0.37	3.55	±	0.46 †,††	3.47	±	0.40 ‡‡	3.35	±	0.39	3.32	±	0.14	Fear > Happiness, Neutral, Sadness, or Disgust Happiness > Neutral, Sadness, or Disgust

**: *p* < 0.01, Happiness vs. Fear or Neutral; ##: *p* < 0.01, Fear vs. Disgust, Happiness, or Sadness; †: *p* < 0.05, Fear vs. Happiness; ††: *p* < 0.01, Fear vs. Happiness, Neutral, Sadness, or Disgust; ‡‡: *p* < 0.01, Happiness vs. Neutral, Sadness, or Disgust. *n.s.*, not significant.

**Table 2:** Descriptive Statistics of Normalized GSR (Z-scores) across Conditions (n = 37).

Condition	M	SD	Min	Max
Disgust	−0.38	0.89	−1.95	1.51
Fear	0.08	1.09	−1.79	1.75
Happiness	−0.17	1.00	−1.95	1.48
Sadness	0.17	1.07	−1.91	1.92
Neutral	0.04	0.88	−1.58	1.54

SD = Standard Deviation; Min = Minimum; Max = Maximum.

**Table 3:** The eye-tracking metric for the five categories of emotional stimuli separately for the low-anxiety, moderate-anxiety, and high-anxiety groups.

	Disgust			Fear			Happiness			Sadness			Neutral			Significant Comparisons
Low anxiety group																
Total fixation duration	2479.20	±	545.06	2322.95	±	361.07	2715.42	±	372.69	2672.13	±	295.26	2702.58	±	361.03	*n.s.*
Average fixation duration	308.85	±	89.29	384.75	±	106.60	298.80	±	65.03	317.78	±	51.68	396.65	±	167.33	*n.s.*
Fixation count	8.33	±	1.71	7.10	±	1.52 *	9.53	±	1.70	8.93	±	1.85	8.23	±	2.28	Fear < Happiness
First fixation duration	224.95	±	58.29	308.33	±	125.52	196.23	±	44.12	222.10	±	68.35	332.53	±	214.01	*n.s.*
Mean pupil diameter during fixation	3.45	±	0.45	3.77	±	0.52 *	3.63	±	0.47 †	3.48	±	0.45	3.43	±	0.46	Fear > Sadness or Disgust or Neutral Happiness > Neutral
Moderate anxiety group																
Total fixation duration	2659.99	±	363.28	2590.97	±	386.54	2675.49	±	472.61	2855.94	±	221.66	2583.34	±	396.13	*n.s.*
Average fixation duration	340.97	±	119.79	395.23	±	107.51 *	303.24	±	52.10	330.70	±	50.31	391.05	±	153.58	Fear > Happiness
Fixation count	8.89	±	2.01	7.67	±	1.92 *	9.40	±	2.02	9.18	±	1.13	8.17	±	2.25	Fear < Happiness or Sadness
First fixation duration	250.69	±	117.36	267.66	±	179.97	242.86	±	69.65	264.27	±	97.74	284.20	±	172.89	*n.s.*
Mean pupil diameter during fixation	3.23	±	0.29	3.38	±	0.36 *	3.31	±	0.26	3.19	±	0.28	3.17	±	0.373	Fear > Sadness or Disgust or Neutral
High anxiety group																
Total fixation duration	2776.91	±	470.87	2602.00	±	582.27	2757.15	±	281.38	2712.73	±	392.15	2564.47	±	452.37	*n.s.*
Average fixation duration	371.89	±	143.38	392.91	±	60.5 *	330.63	±	52.81	374.57	±	185.55	411.32	±	151.56	Fear > Happiness
Fixation count	8.37	±	2.11	7.07	±	2.05 *	8.85	±	1.57	8.39	±	2.12	7.48	±	2.05	Fear < Happiness
First fixation duration	309.69	±	120.15	290.40	±	110.17	243.55	±	42.26	321.93	±	290.15	319.29	±	156.96	*n.s.*
Mean pupil diameter during fixation	3.39	±	0.39	3.59	±	0.47 *	3.52	±	0.43	3.43	±	0.43	3.40	±	0.42	Fear > Sadness or Disgust or Neutral

*: *p* < 0.05 Fear vs. Happiness (or other specific emotions as indicated in the Significant Comparisons column.); †: *p* < 0.05 Happiness vs. Neutral. *n.s.* = not significant.

**Table 4:** Descriptive Statistics of Normalized GSR (Z-scores) across Groups for Each Measure.

Measure	Group	N	Mean	SD	Min	Max
Disgust	Low-anxiety group	15	−0.44	1.02	−1.95	1.49
	Moderate-anxiety group	14	−0.32	0.87	−1.60	1.51
	High-anxiety group	8	−0.35	0.72	−1.39	1.11
Fear	Low-anxiety group	15	0.06	1.16	−1.68	1.37
	Moderate-anxiety group	14	0.11	1.15	−1.40	1.75
	High-anxiety group	8	0.06	0.99	−1.79	1.42
Happiness	Low-anxiety group	15	−0.16	0.74	−1.87	0.76
	Moderate-anxiety group	14	−0.16	1.03	−1.68	1.48
	High-anxiety group	8	−0.21	1.44	−1.95	1.38
Sadness	Low-anxiety group	15	0.17	1.01	−1.61	1.92
	Moderate-anxiety group	14	0.65	1.07	−1.91	1.86
	High-anxiety group	8	−0.65	0.66	−1.65	0.31
Neutral	Low-anxiety group	15	−0.02	0.87	−1.54	1.19
	Moderate-anxiety group	14	−0.01	1.01	−1.58	1.54
	High-anxiety group	8	0.25	0.73	−0.79	1.37

SD = Standard Deviation; Min = Minimum; Max = Maximum.

## Data Availability

The data supporting the findings of this study are available from the corresponding author upon reasonable request. Due to privacy and ethical restrictions, not all data can be publicly shared. However, anonymized data can be provided for academic and research purposes.

## Abbreviations

The following abbreviations are used in this manuscript:

ANOVA	Analysis of variance
GSR	Galvanic skin response
IAPS	International Affective Picture System
STAI	State-Trait Anxiety Inventory
